# The Functionality Assessment Flowchart (FAF): a new simple and reliable method to measure performance status with a high percentage of agreement between observers

**DOI:** 10.1186/s12885-015-1526-0

**Published:** 2015-07-05

**Authors:** Carlos Eduardo Paiva, Felipe Augusto Ferreira Siquelli, Henrique Amorim Santos, Marina Moreira Costa, Daniella Ramone Massaro, Domício Carvalho Lacerda, João Soares Nunes, Cristiano de Pádua Souza, Bianca Sakamoto Ribeiro Paiva

**Affiliations:** 1 Department of Clinical Oncology, Barretos Cancer Hospital, Pio XII Foundation, Barretos, São Paulo Brazil; 2 Health-Related Quality of Life Research Group (GPQual), Barretos Cancer Hospital, Pio XII Foundation, Barretos, São Paulo Brazil; 3 Center for Research Support - NAP, Barretos Cancer Hospital, Pio XII Foundation, Barretos, São Paulo Brazil; 4 Barretos School of Health Sciences, Dr. Paulo Prata - FACISB, Barretos, São Paulo Brazil; 5 Departamento de Oncologia Clínica, Divisão de Mama e Ginecologia, Rua Antenor Duarte Vilella, 1331, Bairro Dr Paulo Prata, CEP: 14784-400 Barretos, SP Brazil

**Keywords:** Performance status, Cancer, Validity, Scales, Assessment

## Abstract

**Background:**

Performance status (PS) assessment is an integral part of the decision-making process in cancer care. Karnofsky Performance Status (KPS) and Eastern Cooperative Oncology Group (ECOG) PS are the most widely used tools. In some studies, the absolute agreement rate of these tools between observers has been moderate to low. The present study aimed to evaluate the inter-observer reliability and construct validity of the new Functionality Assessment Flowchart (FAF) and compare it with ECOG PS and KPS in a sample of cancer patients.

**Methods:**

The patients were recruited by convenience from the waiting rooms of the Breast and Gynecology Ambulatory in a cross-sectional study. Two trained medical students (observer A) and five medical oncologists (observers B) independently rated women according to the ECOG PS, KPS and FAF. After the determining the PS scores, observer A administered the Functional Assessment of Cancer Therapy-Fatigue (FACT-F) questionnaire to the participants. The agreements between observers A and B were investigated using the absolute agreement rate (%), weighted and unweighted kappa and Spearman’s correlation test. For construct validity, the PS scores were correlated with functional and fatigue scores by performing correlation analysis.

**Results:**

Eighty women with a median age of 57 years were included in the study (86 % accrual rate). Among these women, 39 (48.8 %) had advanced cancer. The overall absolute agreement rate between observers was 49.4 % for KPS, 67 % for ECOG PS, and 78.2 % for FAF. When using unweighted kappa values, the inter-observer reliability was “fair”, “moderate” and “substantial” for KPS, ECOG PS and FAF, respectively. However, when using weighted kappa statistics, “substantial” agreement was observed for KPS and ECOG PS and “nearly perfect” agreement was observed for FAF. All of the PS scales correlated very well with the functional and fatigue scores.

**Conclusions:**

We present a new instrument with moderate to high inter-observer agreement and adequate construct validity to measure PS in cancer patients.

**Electronic supplementary material:**

The online version of this article (doi:10.1186/s12885-015-1526-0) contains supplementary material, which is available to authorized users.

## Background

Performance status (PS) is an assessment of the patients’ actual level of function, ability for self-care and level of ambulation [1]. PS scales are used as selection criteria and for the stratification of subgroups in clinical trials. They are also used to evaluate the impact of cancer treatments on health-related quality of life and as an outcome measure to compare differences in the functional performance before and after exposure to a specific therapy [2]. Moreover, a patient’s PS score is widely used as an aid in the decision to receive anticancer treatment or palliative care only [3].

The Karnofsky Performance Status (KPS) was introduced in 1949 by Karnofsky and Burchenal [4] as an 11-point measure of the functional status, ranging from 0 % (death) to 100 % (normal functioning). The Eastern Cooperative Oncology Group (ECOG) PS was developed as an alternative and easier PS assessment tool [5]. By having fewer response options (from 0 to 5), the ECOG PS is better than KPS in terms of inter-observer agreement; however, the ECOG PS likely did not retain the ability to more comprehensively detail a patient’s PS [6]. The Palliative Performance Scale (PPS) was proposed in 1996 to measure the PS of patients undergoing palliative care [7]. The PPS was created as an alternative to KPS in an attempt to improve the assessment of PS of low-functional palliative-care patients. Among the PS evaluation scales in oncology, the KPS, ECOG PS and more recently, PPS are the most widely used [8].

Although these scales are widely used in the clinical decision-making process in practice and research settings, information on inter-observer agreement is scarce and mostly dates from the 1980s. Regarding the rates of absolute agreement between the raters, recent papers have reported contradictory findings [1, 9]. Moderate to high concordance rates were found for KPS (63–75 %) and ECOG PS (90–92 %) in a study that included patients with better-functioning scores [1]; however, another study [9] found low absolute agreement rates in a palliative care setting (ECOG PS = 53–61 %; KPS = 38–50 %). Therefore, there is a need for the development of new valid scales or assessment strategies showing better inter-observer reliability. Previously, other authors [3] developed an algorithm to more objectively measure PS based on KPS. We used their work as a basic foundation for developing our new strategy to evaluate PS using a flowchart. Unlike the aforementioned study, the Functionality Assessment Flowchart (FAF) considers some patients’ responses and was developed based on the fundamental aspects not only of the KPS, but also of the ECOG PS and PPS. Our hypothesis was that the FAF, by containing patients’ opinions, would yield a higher inter-observer reliability than other PS scales with similar construct validity.

This preliminary study aimed to assess the PS of patients with cancer using the FAF and evaluate the agreement of scores measured by two independent raters. Moreover, the agreement of FAF between observers and its correlation with the functionality and fatigue scores were compared with the results of the ECOG PS and KPS.

## Methods

### Study design and setting

A cross-sectional study was conducted in the Barretos Cancer Hospital (Barretos, SP, Brazil). The patients were recruited from the waiting rooms of the Breast and Gynecology ambulatory.

### Ethics statement

The local Research Ethics Committee approved the present study (no. 644.297). In compliance with the Declaration of Helsinki and Resolution 466/12 of the Brazilian National Health Council, which addresses research on human beings, the study aims were explained to the participants, who then provided informed consent.

### Development of the Functionality Assessment Flowchart (FAF)

**Fig. 1:**
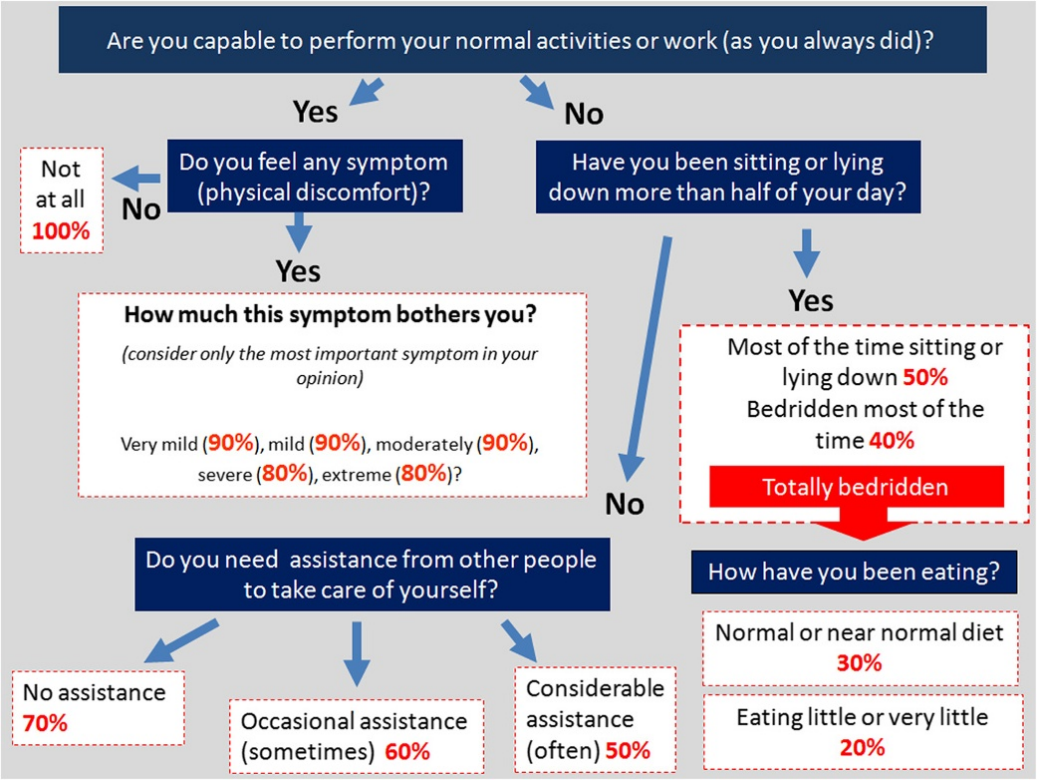
English version of Functionality Assessment Flowchart (FAF). The questions are shown inside the blue squares. Responses are driven according to the arrow direction as a flowchart. Final evaluation of performance status is shown in red numbers as percentage values

A detailed revision of the ECOG-PS, KPS and PPS was performed by the authors to use pieces from each performance status scale for the construction of a hybrid tool that considers the patients’ opinions about their own functionality. The authors conducted several meetings to discuss instrument drafts until a final version was considered adequate for testing. The FAF was designed for systematic administration by an interviewer and as a flowchart. The questions are highlighted in blue; the flowchart ends after reaching any percentage. The English version of the instrument is shown in Fig. 1 and the original Portuguese version in shown as Supplementary Material (see Additional file 1).

### Observers

Two medical graduate students and 5 medical oncologists participated in the study as observers. All of the participants received printed scales and information regarding the correct method to use the scales. Of note, the medical graduate students were trained to evaluate the patient’s PS using clinical simulated vignettes and then observing one of the authors (CEP) in medical consults for two consecutive weeks. High agreement rates between medical graduate students and the advisor were not considered a prerequisite for closing the pre-study training. Nevertheless, it were required that the students should memorize the scales; demonstrate familiarity with them; and present logical explanations to justify every chosen PS category. After reaching these criteria, the medical students should be checked in additional 10 evaluations maintaining the same standard to be considered ready to perform the study assessments.

### Data collection

The observers were coded as observers A or B depending on personal availability. Observer A was always a trained medical student, and observer B was a medical oncologist; both of the observers evaluated patients using the ECOG-PS, KPS and FAF. The evaluations were independent, and the scales were used in a random sequence. The Functional Assessment of Cancer Therapy-Fatigue (FACT-F) questionnaire was applied by observer A only after defining the PS score. Patients unable to answer the FACT-F questionnaire were evaluated only regarding PS; in these cases, the FAF was answered using information provided by the caregivers.

### Instruments

The FACT-F questionnaire was specifically developed to measure fatigue associated with anemia in cancer populations [10]. The FACT-F is a valid Brazilian, 40-item instrument that contains the 27 items of FACT-G (subdivided into four primary domains of quality of life: physical well being, social and family well being, emotional well being, and functional well being) and 13 fatigue-related questions [11]. In patients with cancer, the Functional Assessment of Chronic Therapy-Fatigue (FACT-F) scale can differentiate patients by hemoglobin level and patient-rated performance status [12]. In the present study, we decided a priori to use the functional well being scale (FWB) (range: 0–28), the fatigue subscale (FS) (range: 0–52) and the FACT-F Trial Outcome Index (TOI) (range: 0–108) as indicators of functionality. Higher the scores indicated better functionally.

ECOG-PS is a measure of PS that ranges from 0 (fully active) to 5 (dead) [5]. The KPS ranges from 100 % (normal) to 0 % (dead) [4]. Translated Brazilian versions of the ECOG-PS and KPS were used in the study. All of the instruments were used in paper-and-pencil form.

### Sample size estimation

The sample size was estimated considering 60 % and 85 % concordance rates for the KPS and FAF, respectively. Using a significance level of 5 % for alpha and 20 % for beta, the sample size that was required for this preliminary study was 76 patients.

### Statistic analysis

Correlations were analyzed using Spearman’s rank correlation coefficient. The concordance pattern was evaluated using both the unweighted and the weighted kappa statistics; the strength of agreement was as follows: <0.00 = poor agreement, 0.00–0.20 = slight agreement, 0.21–0.40 = fair agreement, 0.41–0.60 = moderate agreement, 0.61–0.80 = substantial agreement, and 0.81–1.00 = nearly perfect agreement [13]. The adopted significance level was 0.05. The statistical softwares used were SPSS version 20.0 (SPSS; Chicago, IL, USA) and MedCalc Statistical Software version 14.8.1 (MedCalc Software bvba, Ostend, Belgium).

## Results

### Sample characteristics

Between February 2014 and August 2014, 86 women were invited to participate in the study. Of these women, 6 refused to participate due to extreme fatigue. Among the 80 women included in the study, 10 did not complete the FACT-F due to poor clinical conditions.

The median age was 57 years (range, 30–80). Thirty-six (*n* = 36, 45 %) women were married, 38 (47.5 %) were studied for less than 8 years, and the majority (*n* = 60, 75.9 %) were inactive. The main primary tumor sites were the breast (*n* = 55, 68.8 %), uterine cervix (*n* = 14, 17.5 %) and ovary (*n* = 4, 5 %). Thirty-nine (*n* = 39, 48.8 %) patients received some type of palliative therapy for advanced cancer. Table 1 describes the primary socio-demographic and clinical characteristics of the evaluated patients.

**Table 1:** Clinical and sociodemographic characteristics of the patients (*n* = 80)

Characteristics	N (%)
Age (years)	
Median (range)	57.0 (30–80)
Mean (SD)	57.3 (11.9)
Marital status	
Married	36 (45)
Divorced	10 (12.5)
Single	14 (17.5)
Widowed	19 (23.8)
Years of formal education	
Illiterate	10 (12.8)
Less than 8	28 (35.9)
8–11	28 (35.9)
Higher than 11	12 (15.4)
Unknown	2
Work activities	
Active	19 (24.1)
Inactive	60 (75.9)
Unknown	1
Primary tumor sites	
Breast	55 (68.8)
Cervix	14 (17.5)
Ovarian	4 (5.0)
Endometrial	3 (3.8)
Vulvo-vaginal	4 (5.0)
Distant metastasis	
Yes	39 (48.8)
No	41 (51.2)
Actual treatment	
NED/follow-up	7 (8.8)
Adjuvant/neoadj chemotherapy	13 (16.3)
Adjuvant hormone therapy	17 (21.3)
Adjuvant trastuzumab	3 (3.8)
Palliative chemotherapy	28 (35.0)
Palliative hormone therapy	9 (11.3)
Radio chemotherapy	1 (1.3)
Palliative care only	2 (2.5)

*SD* standard deviation, *NED* no evidence of disease, *Neoadj* neoadjuvant

### Agreement between observers’ analyses

The overall absolute agreement rate between the observers was 49.4 % (39 of 79) for the KPS, 67 % (53 of 79) for the ECOG PS, and 78.2 % (61 of 78) for the FAF. A comparison between the proportions indicated that FAF presented a higher rate of agreement than the KPS (Table 2). When using unweighted kappa values, inter-observer reliability was “fair”, “moderate” and “substantial” for KPS, ECOG PS and FAF, respectively. However, when using weighted kappa values, the inter-observer reliability results improved significantly, reaching substantial agreement for KPS and ECOG PS and nearly perfect agreement for FAF (Table 2). All of the KPS, ECOG PS and FAF pairings were highly significantly correlated, with correlation coefficients of approximately 0.9 (Table 2).

**Table 2:** Agreement analyses between different observers of the ECOG PS, KPS and FAF

PS Scales	Agreement* (%) (95 % CI)	Unweighted kappa (95 % CI)	Weighted kappa (95 % CI)	Spearman’s correlation (95 % CI)
ECOG-PS	67.0 (50.0–88.0) ^a, b^	0.561 (0.427–0.695) ^1^	0.763 (0.679–0.847) ^3^	0.890 (0.833–0.928)
KPS	49.4 (35.1–67.5) ^b^	0.396 (0.272–0.520) ^2^	0.747 (0.672–0.822) ^3^	0.905 (0.855–0.938)
FAF	78.2 (59.8–100) ^a^	0.709 (0.600–0.819) ^3^	0.826 (0.741–0.911) ^4^	0.893 (0.837–0.930)

*Overall absolute agreement rate. Different letters indicate significant results (ECOG-PS versus KPS, *p* = 0.144; ECOG-PS versus FAF, *p* = 0.413; KPS versus FAF, *p* = 0.023). ^1^ Moderate agreement; ^2^ fair agreement; ^3^ substantial agreement; ^4^ nearly perfect agreement

### Construct validity analyses

In general, the correlation coefficients between the FAF and the FWB, FS and TOI scores were slightly higher than those between the other PS scales with the FWB, FS and TOI scores. However, all of the coefficients presented overlapping 95 % confidence intervals and should thus be considered similar (Table 3).

**Table 3:** Spearman correlation analyses between performance status scores and functionality and fatigue scores from FACT-F

Correlation coefficients (95 % CI)			
Domain	ECOG-PS	KPS	FAF
FWB	−0.640 (−0.727; −0.532)	0.656 (0.553; 0.741)	0.672 (0.583; 0.750)
FS	−0.499 (−0.625; −0.344)	0.538 (0.392; 0.656)	0.574 (0.435; 0.676)
TOI	−0.606 (−0.714;-0.472)	0.639 (0.509; 0.736)	0.680 (0.569; 0.756)

*FWB* functional wellbeing, *FS* fatigue subscale, *TOI* trial outcome index

The results were significant at <0.001

## Discussion

Cancer treatments are initiated and terminated based on PS scores; inaccurate estimates may lead to a failure to receive treatment that may be helpful or to a patient receiving an aggressive treatment that should have been avoided. Moreover, the PS is largely used to select participants for inclusion in clinical trials. Thus, PS assessment is an essential part of oncological care and must be evaluated with high accuracy levels. In the present study, we present a simple and reliable flowchart that considers patient opinions and that demonstrates high absolute concordance rates and good construct validity.

The FAF is a new method to evaluate the PS of patients with cancer, compensating for the lack of instruments to measure functionality in detail (on an 11-point scale) with a high concordance rate between observers. The absolute concordance rate in the present study yielded nearly 80 % agreement, which was much higher than the absolute agreement of the KPS (~50 %) and ECOG-PS (67 %). Regarding the ECOG-PS, previous studies found absolute agreement ranging from 40 % to 93 % [1, 9, 14, 15]. The inter-observer variability increases as the number of choice increases [6]. Thus, the absolute agreement rate of the KPS between observers is generally lower than that of ECOG-PS, varying from 38 % to 76 % [1, 2, 9, 15].

Previous studies evaluated the agreement rates between observers by performing correlation analyses. In general, high correlation coefficients (*r* > 0.80) have been observed for ECOG-PS and KPS [2, 9, 16]. In accordance with previous studies, we found Spearman correlation coefficients of approximately 0.9 for all three of the evaluated scales. Moreover, our study highlights that high correlation levels are not necessarily associated with high agreement between raters.

Although the overall percentage of agreement provides a measure of agreement, it does not consider the agreement that would be expected purely by chance. The kappa statistic, however, is a measure of “true” agreement [17]. We found a clearly higher value of the kappa statistic for FAF compared with that for KPS. However, considering that our instruments are all ordinal multi-category scales, kappa can be weighted to confer greater importance to large differences than small differences between ratings. The KPS and FAF weighted kappa values were similar, suggesting that the disagreements between observers regarding KPS were primary small differences. The same pattern of improvement in agreement values from unweighted to weighted kappa were also observed by Meyers et al. [9].

One advantage of the FAF over the other tested scales is that it considers the patient’s opinion about their own functional states. As we hypothesized, the FAF can improve the concordance rates between raters. However, some women could have inaccurately answered the first step of the FAF (“Are you able to work or to do your daily activities?”), causing secondary gains by considering themselves worse (leave or absence from work due to illness) or better (as a way to feel more optimistic) than they actually were. FAF raters must understand that the FAF is a flowchart developed to facilitate PS evaluation and not a rigid measure based strictly on patient responses.

The lack of a functional gold standard tool was a challenge for this study. Thus, to evaluate the construct validity of the FAF, we compared its scores with functional and fatigue scores obtained from the previously validated Brazilian version of the FACT-F questionnaire [11]. As expected, the correlation between the functional and fatigue scores and the PS scales was strong. Therefore, in terms of construct validity, the FAF should be considered as valid as ECOG-PS and KPS.

### Study limitations

This study was preliminary; therefore, one limitation was its small sample size. Another significant limitation is that all of the study assessments were performed repeatedly at the same ambulatory setting. Only female participants were included, which potentially reduces the generalizability of our results. Although we analyzed many low-functioning participants selected from the waiting rooms, future studies should include a greater sample of both outpatients and inpatients.

### Future perspectives

Our preliminary findings support a subsequent study with a larger and heterogeneous sample size to more definitively investigate the benefit of implementing a PS assessment using the FAF in clinical practice. We are currently developing a computational software containing the FAF and intend to assess its construct validity by comparing its values with more precise functional activity levels measured by digital accelerometers [18]. We consider both the ECOG-PS and KPS to be well-established tools in the oncology setting. However, the FAF has the advantage of evaluating the PS in a more discriminative manner than the ECOG-PS and with a higher concordance rate than KPS. Thus, the FAF is a new tool that requires further refinement and investigation.

## Conclusions

We present a new simple and reliable instrument to measure the PS in cancer patients. The FAF demonstrated good inter-observer agreement and adequate construct validity. The FAF is a potential new tool to assess the PS with high agreement between observers. Further studies are necessary to investigate the FAF in other settings using more-practical computational software.

## Additional file

Additional file 1:
**Original version (Portuguese from Brazil) version of Functionality Assessment Flowchart (FAF).**
