# Supplementary material for: The Functionality Assessment Flowchart (FAF): a new simple and reliable method to measure performance status with a high percentage of agreement between observers
Source: BMC Cancer. 2015 Jul 5;15:501. doi: 10.1186/s12885-015-1526-0 (PMC4491220; doi:10.1186/s12885-015-1526-0)
Supplement: Additional file 1: — Original version (Portuguese from Brazil) version of Functionality Assessment Flowchart (FAF). [file 12885_2015_1526_MOESM1_ESM.pdf]

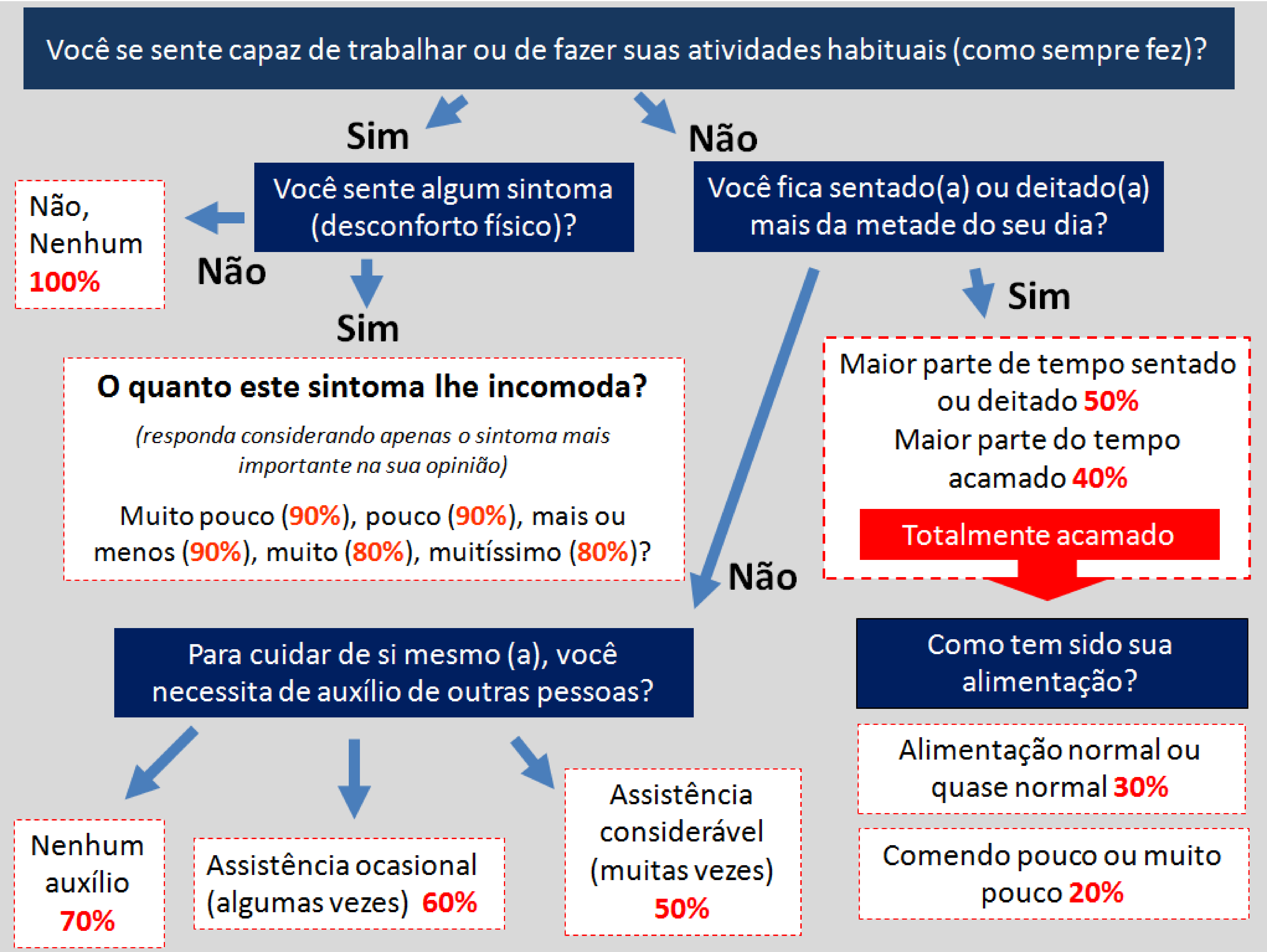

Você se sente capaz de trabalhar ou de fazer suas atividades habituais (como sempre fez)?
Sim
Não
Você sente algum sintoma (desconforto físico)?
Não, Nenhum 100%
Não
Sim
O quanto este sintoma lhe incomoda?
(responda considerando apenas o sintoma mais importante na sua opinião)
Muito pouco (90%), pouco (90%), mais ou menos (90%), muito (80%), muitíssimo (80%)?
Você fica sentado(a) ou deitado(a) mais da metade do seu dia?
Sim
Maior parte de tempo sentado ou deitado 50%
Maior parte do tempo acamado 40%
Totalmente acamado
Não
Como tem sido sua alimentação?
Alimentação normal ou quase normal 30%
Comendo pouco ou muito pouco 20%
Para cuidar de si mesmo (a), você necessita de auxílio de outras pessoas?
Nenhum auxílio 70%
Assistência ocasional (algumas vezes) 60%
Assistência considerável (muitas vezes) 50%
